# 17*β*-Estradiol Promotes Schwann Cell Proliferation and Differentiation, Accelerating Early Remyelination in a Mouse Peripheral Nerve Injury Model

**DOI:** 10.1155/2016/7891202

**Published:** 2016-10-30

**Authors:** Yan Chen, Wenjie Guo, Liangzhi Xu, Wenjuan Li, Meng Cheng, Ying Hu, Wenming Xu

**Affiliations:** ^1^Department of Obstetrics and Gynecology, West China Second University Hospital, Sichuan University, Chengdu 610041, China; ^2^Key Laboratory of Birth Defects and Related Diseases of Women and Children (Sichuan University), Ministry of Education, Chengdu 610041, China; ^3^Department of Anatomy, Chengdu University, Chengdu 610106, China; ^4^The Joint Laboratory for Reproductive Medicine of Sichuan University-The Chinese University of Hong Kong, West China Second University Hospital, Sichuan University, Chengdu 610041, China

## Abstract

Estrogen induces oligodendrocyte remyelination in response to demyelination in the central nervous system. Our objective was to determine the effects of 17*β*-estradiol (E2) on Schwann cell function and peripheral nerve remyelination after injury. Adult male C57BL/6J mice were used to prepare the sciatic nerve transection injury model and were randomly categorized into control and E2 groups. To study myelination in vitro, dorsal root ganglion (DRG) explant culture was prepared using 13.5-day-old mouse embryos. Primary Schwann cells were isolated from the sciatic nerves of 1- to 3-day-old Sprague–Dawley rats. Immunostaining for myelin basic protein (MBP) expression and toluidine blue staining for myelin sheaths demonstrated that E2 treatment accelerates early remyelination in the “nerve bridge” region between the proximal and distal stumps of the transection injury site in the mouse sciatic nerve. The 5-bromo-2′-deoxyuridine incorporation assay revealed that E2 promotes Schwann cell proliferation in the bridge region and in the primary culture, which is blocked using AKT inhibitor MK2206. The in vitro myelination in the DRG explant culture determined showed that the MBP expression in the E2-treated group is higher than that in the control group. These results show that E2 promotes Schwann cell proliferation and myelination depending on AKT activation.

## 1. Introduction

Remyelination is an important aspect of nerve regeneration. Sciatic nerve crush injury model is often used to study peripheral nerve remyelination because the crushed nerve always fully restores structure and function, despite seldom undergoing “abnormal fix” after injury [1, 2]. The peripheral nerve remyelination mainly recapitulates Schwann cell myelination in developmental stages [3–5]. However, developmental peripheral nerve myelination depends on extensive Schwann cell precursor proliferation [6–8],while remyelination in the distal stump of the crushed nerve seems to be independent of the proliferation of Schwann cell precursors generated during Wallerian degeneration, but it rather relies on the existence of the original nonproliferating Schwann cells [9]. In the nerve transection model, the sciatic nerve was severed, and a gap without any tissue was left between the proximal and distal stumps of the cut site because of the elastic recoil of nerve tissue. In a short time, the gap was occupied by a large number of newly emerging Schwann cells and fibroblasts, which were neatly arrayed along the longitudinal axis of the nerve, with growing axons extending from the proximal stump. This gap filled in by newly emerged cells and axons is also known as the “nerve bridge” [10, 11]. Remyelination in the “nerve bridge” may be difficult to attribute to the originally existing Schwann cells because the site is entirely occupied by newly generated Schwann cells and axons and is actually not the zone of Wallerian degeneration [10–12]. So, studying remyelination in the “nerve bridge” may exclude the interference of the Wallerian degeneration process and help researchers understand the process of early remyelination after peripheral nerve injury.

Among the factors promoting remyelination, female hormones (estrogen and progesterone) have recently attracted considerable attention. Clinical and laboratory studies suggest that estrogen and progesterone may have potential therapeutic effects on multiple sclerosis (MS), which is a severe demyelinating disease of the CNS [13, 14]. A previous study also found that progesterone can promote peripheral nerve remyelination [15], probably through the rapid upregulation of* Krox20*, which is the key transcription factor for the formation of myelin. Progesterone also increases the transcription of myelin components such as myelin protein zero (MPZ) and peripheral myelin protein-22 (PMP22) expression in Schwann cells [16–18]. However, limited information is available about the function of estrogen in peripheral nerve remyelination, particularly due to the lack of in vivo studies. The PI3K/AKT/mTOR signaling pathway has been considered as the mechanism behind the promotion of remyelination by estrogen. The PI3K/AKT/mTOR signaling pathway is required for both Schwann cell and oligodendrocyte myelination [19–21] and likely promotes the myelin repair process [21, 22]. Estrogen is considered an AKT activator without direct genomic effects, which is an important mechanism of estrogen-dependent tumorigenesis [23–25]. Therefore, the effect of estrogen on AKT activation may also contribute to myelination and remyelination. In fact, both estrogen and progesterone were found to increase the phosphorylated AKT level in oligodendrocytes, whereas androgen had the opposite effect [26, 27], which might reveal the internal mechanism of female hormones in promoting myelin regeneration by affecting myelin-forming cells. Recent studies have also shown that selective estrogen receptor *β* agonists can activate the PI3K/AKT/mTOR signaling pathway in oligodendrocytes and promote remyelination in the CNS of mice with experimental autoimmune encephalomyelitis [28]. However, whether estrogen promotes peripheral nerve myelination and remyelination via the AKT signaling pathway remains unknown. The objective of this study was to determine the effects of 17*β*-estradiol (E2), a natural form of estrogen, on Schwann cell function and peripheral nerve remyelination after injury. This study found that estrogen may affect differentiation and myelination of Schwann cell via the AKT/mTOR signaling pathway, thereby promoting peripheral nerve remyelination after peripheral nerve injury.

## 2. Materials and Methods

### 2.1. Animals

Adult male C57BL/6J mice (aged 8 weeks; specific pathogen-free grade) and 1- to 3-day-old Sprague–Dawley rats (specific pathogen-free grade) were purchased from the Experimental Animal Center of the People's Hospital in Sichuan Province, China. E13.5 of the fetal rat was obtained under the condition of SPF feeding. All animal experiments were performed in accordance with the National Institutes of Health Guide for the Care and Use of Laboratory Animals (NIH Publication Number 8023, revised 1978) and approved by the Ethics Committee of Sichuan University.

### 2.2. Sciatic Nerve Transection Injury Model

**Figure 1:**
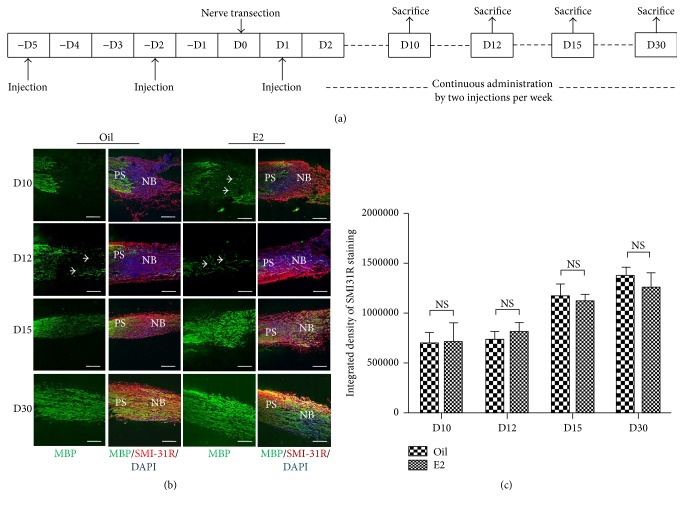
E2 treatment accelerated early expression of MBP in the nerve bridge upon sciatic nerve transection. (a) A schematic drawing showing the experimental setting. The mice were injected with E2 or solvent 5 days prior to nerve transection (−D5*～*−D1), and two injections per week were continued for a sustained drug delivery till the mice were sacrificed. The surgery was carried out at D0, and the mice were sacrificed for sample collection at D10, D12, D15, and D30, respectively, after the surgery. (b) Immunofluorescence images of expression of MBP (green signals, white arrow) in the longitudinal section of the nerve bridge (NB) from 10, 12, 15, and 30 day after injury are shown individually. NF staining (red signals) was used to illustrate axons, and some proximal stump (PS) where the MBP signal was regular and strong was retained left of the nerve to distinguish the site of the NB, which is adjacent. Scale bars represent 250 *μ*M (100x). MBP, myelin basic protein; NF, neurofilament; PS, proximal stump. (c) Quantification of axonal signal SMI-31R (*n* = 5; NS, no significance).

To prepare the sciatic nerve transection injury model, C57BL/6J mice were randomly categorized into control and E2 groups. The left sciatic nerves of the mice from each group were exposed under general anesthesia under aseptic conditions. The left sciatic nerves were transected at the midthigh level. The mice from the E2 group were treated with E2 (Sigma-Aldrich, St. Louis, MO) dissolved in castor oil (1 mg/kg in total amount per injection) 5 days prior to surgery [29, 30]. Two injections per week were continued to prevent a gradual decline of steroid plasma levels in the second half of the week [31] until the mice were sacrificed (Figure 1(a)). The mice from the control group were injected with castor oil only. Radioimmunoassay kit following the manufacturer's protocol (Union Medical & Pharmaceutical Technology, Tianjin, PRC) was used to test E2 levels. The serum level of E2 from the E2 group was 2130.38 ± 600.43 pg/mL, which was significantly higher than that of the control group (12.73 ± 3.24 pg/mL).

### 2.3. Primary Schwann Cell Culture and Drug Administration

Primary Schwann cells were isolated from the sciatic nerves of 1- to 3-day-old Sprague–Dawley rats. For purification, the cells were treated with cytosine arabinoside (10 *μ*M, Sigma-Aldrich) twice for 48 h in Dulbecco's modified Eagle's medium (DMEM, Gibco, Grand Island, NY) supplemented with 10% fetal bovine serum (FBS; Gibco), followed by complement (Calbiochem, Darmstadt, Germany) mediated lysis of fibroblasts using anti-thy1.1 antibody (Serotec, Oxford, UK). Then, the purified Schwann cells were expanded on poly-D-lysine-coated plates in phenol red free DMEM (Gibco, Cat. 31053028) supplemented with 10% FBS stripped of charcoal, 5% CO_2_, 37°C, 2.5 *μ*M of forskolin (Sigma-Aldrich), and 10 ng/mL of human *β*1-heregulin (EGF domain; Sigma-Aldrich). To study the effect of hormone on mRNA expression of Schwann cells, primary Schwann cells were maintained in the medium without forskolin and *β*1-heregulin for 24 h prior to the addition of 100 nM E2 [32]. To study the effect of hormone on Schwann cell proliferation, the cells were incubated in a medium with forskolin rather than *β*1-heregulin for 24 h before 100 nM E2 was added [33].

In order to eliminate the influence of the endogenous steroid hormone in the culture medium, phenol red free DMEM and charcoal-stripped FBS were used for the expanding of purified Schwann cells and the later cell culture experiment for investigating of E2 function. To produce charcoal-stripped serum, FBS was briefly incubated with dextran-coated charcoal (Sigma-Aldrich, 2 g in 100 mL of FBS) and was mixed gently on a shaker table at 4°C overnight. Later, the charcoal was removed by centrifuging the suspension at 2000 g for 15 min. The supernatant was filtered through a 0.45 *μ*M filter unit (Millipore, Bedford, MA).

### 2.4. DRG Explant Culture and Drug Administration

To study myelination in vitro, DRG explant culture [34] was prepared using 13.5-day-old mouse embryos (embryo day 13.5, E13.5). After dissection, the DRGs were seeded on collagen- (Sigma-Aldrich) coated coverslips without enzyme digestion. Axonal processes and endogenous Schwann cells were allowed to grow and establish fine networks for 1 week in phenol red free DMEM supplemented with 10% charcoal-stripped FBS and 100 ng/mL nerve growth factor (Roche, Basel, Switzerland). Later, ascorbic acid (50 *μ*g/mL; Sigma-Aldrich) was added to induce myelination for another week. To study the effect of hormone on in vitro myelination, 100 nM E2 and ascorbic acid were simultaneously added to the DRG explant culture. For the pathway study, 100 nM of MK2206 (AKT inhibitor; Selleckchem, Houston, TX) was added 1 h prior to E2 administration. All drugs were maintained in the culture medium until the cells were collected for assay.

### 2.5. Immunofluorescence

Mice were anesthetized using ether and then intracardially perfused with 0.01 M phosphate-buffered saline (PBS), followed by 4% paraformaldehyde (PFA). The sciatic nerves were removed and postfixed in 4% PFA for 30 min and then cryoprotected by immersion in 25% sucrose, embedded with OCT compound, and 6-*μ*M sections were cut in Leica CM3050S cryostat microtome. Cells grown on coverslips were fixed in 4% PFA for 10 min. Tissue sections or cells were blocked with PBS containing 0.3% Triton X-100 and 5% FBS and incubated with certain primary antibodies at 4°C overnight and with secondary antibodies at room temperature for 2 h. Finally, tissues were counterstained with 4′,6-diamidino-2-phenylindole (DAPI) to determine the nuclei before mounting. The results were acquired using laser confocal microscopy (Olympus FV1000; Olympus, Tokyo, Japan) and fluorescence microscopy (Nikon Eclipse Ti-U; Nikon, Tokyo, Japan). The primary antibodies used were as follows: goat anti-Sox10 polyclonal antibody (1 : 200; Santa Cruz, Dallas, TX), rabbit anti-c-Jun monoclonal antibody (1 : 200, Epitomics, Burlingame, CA), mouse anti-BrdU monoclonal antibody (1 : 500, BD), rabbit anti-myelin basic protein (MBP) polyclonal antibody (1 : 500; Abcam; Cambridge, UK), SMI-31R (1 : 1000; Covance, Princeton, NJ), mouse anti-S100*β* monoclonal antibody (1 : 500; Sigma-Adrich), rabbit anti-phosphorylated S6 monoclonal antibody (1 : 500; Cell Signaling, Boston, MA), rabbit anti-Ki67 polyclonal antibody (1 : 200; Thermo, Waltham, MA), and rabbit anti-P75 polyclonal antibody (1 : 400; Millipore). The Cy3, Cy2, or Alexa Fluor 488 conjugated secondary antibodies were all purchased from Jackson (Lancaster, PA) and used at 1 : 1000 dilution.

### 2.6. BrdU Incorporation Assay

To study cell proliferation in vivo, mice were injected intraperitoneally with 100 *μ*g of 5-bromo-2′-deoxyuridine (BrdU) per gram of body weight. Two hours after BrdU injection, the sciatic nerves were removed and subjected to cryosection. To study cell proliferation in vitro, primary Schwann cells were incubated with 10 *μ*g/mL of BrdU for 2 h prior to PFA fixation. For BrdU staining, immunofluorescence was performed according to the method described earlier; but as an additional step, tissues or cells were denatured in 2N HCl for 20 min at 37°C before blocking.

### 2.7. Histomorphology

To study the histology of the myelin sheath, the mice were intracardially perfused with 0.2 M of sodium cacodylate before the sciatic nerves were dissected. The nerves were then immersed in 2.5% glutaraldehyde in 0.1 M sodium cacodylate for 2 h and then transferred to 0.1 M of sodium cacodylate buffer with 4% PFA and 2.5% glutaraldehyde, followed by further fixation with 2% osmium tetroxide. The fixed tissue was dehydrated through a graded acetone series, as described earlier [35], and embedded in Spurr's resin to obtain semithin sections. Thereafter, 1 *μ*M sections were acquired and stained with 1% toluidine blue for analysis under a light microscope.

### 2.8. Western Blot

“Nerve bridge” tissues (from four sciatic nerves) or cells were homogenized in radio immunoprecipitation assay buffer (RIPA) 150 mM NaCl, 1% NP-40, 0.5% sodium deoxycholate, 0.1% sodium dodecyl sulfate (SDS, 50 mM Tri-HCl, pH 8.0) with Protease Inhibitor Cocktails (Sigma-Aldrich), phenylmethylsulfonyl fluoride (Amresco, Solon, Cleveland, OH), and protein phosphatase inhibitor cocktail (Sigma-Aldrich). Tissue lysate was processed using standard SDS-polyacrylamide gel electrophoresis and western blot. Highly sensitive chemiluminescence (ECL kit from Millipore) was used to illustrate the bands. Images were obtained through X-ray film exposure system. Densitometric analysis of the bands was performed using NIH Image J software package (http://rsb.info.nih.gov/ij/). Primary antibodies used were as follows: rabbit anti-AKT1 monoclonal antibody (1 : 3000; Cell Signaling), rabbit anti-phosphorylated AKT monoclonal antibody (S473, 1 : 2000; Cell Signaling), rabbit anti-phosphorylated S6 (1 : 2000, Cell Signaling), rabbit anti-phosphorylated mTOR (1 : 1000; Cell Signaling), and rabbit anti-MBP polyclonal antibody (1 : 1000; Abcam). The horseradish peroxidase conjugated secondary antibodies were all purchased from Millipore and used at 1 : 10,000–1 : 50,000 dilution.

### 2.9. RNA Preparation and Quantitative Real-Time Polymerase Chain Reaction (qRT-PCR)

Total RNA was extracted after cells were lysed in TRIzol reagent (Invitrogen, Carlsbad, CA). The concentration of total RNA was measured using a spectrophotometer (Nanovue plus; GE, Fairfield, CT), and 500 ng of RNA from each sample was subjected to reverse transcription using one-step core Prime Script RT reagent kit (Takara, Kusatsu, Japan). cDNA samples were amplified using certain primers in SSO Fast EvaGreen Supermix (Bio-Rad, Hercules, CA) with CFX96™ Real-Time System (Bio-Rad). GAPDH expression was used to normalize target gene expression and to obtain relative expression values that were used to calculate percentage changes. The sequences of the qRT-PCR primers used are as follows (5′-3′):* Krox20*: forward, TTGATCAGATGAACGGAGTGG; reverse, GTGAAGGTCTGGTTTCTAGGC;* Mag*: forward, GCTACAACCAGTACACCTTCTC; reverse, TGACCTCTACTTCCGTTCCTG;* Mpz*: forward, GACAACGGCACTTTCACATG; reverse, GATCACGGCTCCCAACAC.* Sox10*: forward, GCACGCAGAAAGTTAGCC; reverse, TGTCACTCTCGTTCAGCAAC; GAPDH: forward: GATGCTGGTGCTGAGTATGRCG; reverse GTGGTGCAGGATGCATTGCTCTGA.

### 2.10. Statistical Analysis

To facilitate statistical analysis, we included 6 mice in each group at each time point when the mice were sacrificed for the experiments of immunostaining and toluidine blue staining of nerve tissue slices. However, some mice were excluded from the study, because the nerve bridge failed to form or the incision on the skin was of much swelling. Still, we ensured that 5-6 mice were collected for the final tests in each group per time point of treatment. For western blot analysis, we collected 4 nerve bridges for the tissue lysing from each group in order to get enough tissue proteins, and we routinely conducted 3 biological repetitions for statistical analysis.

For all group experiments, measurement data or enumeration data were created using GraphPad Prism 5 software (GraphPad Prism software, Inc., La Jolla, CA) and presented as mean ± standard error of mean. Student's *t*-test was performed using SPSS version 15.0 for Windows (SPSS Inc., Chicago, IL) to test the significance. *P* < 0.05 was considered statistically significant.

## 3. Results

### 3.1. E2 Accelerated Early Remyelination in “Nerve Bridge” of Transected Mouse Sciatic Nerve

To study the effect of E2-accelerated early remyelination in “nerve bridge” of transected mouse sciatic nerve, the mouse model was used. It was found that most “nerve bridges” were not completely formed until 10 days after injury (see Figures S1(A) and S1(B) in Supplementary Material available online at http://dx.doi.org/10.1155/2016/7891202). At 10 days after injury, no visible myelin sheath in the bridge site was found, and newly generated axons seldom underwent degeneration (Figure S1(C)), unlike the distal stump, wherein many degenerated axons and myelin sheaths were found via toluidine blue staining of semithin tissue slice (Figure S1(C)).

To illustrate remyelination, MBP, which is a major component of compact myelin, was stained. In the longitudinal section slices of injured sciatic nerves, minimal expression of MBP was found in the bridge site of control sciatic nerves, whereas sporadic signals of MBP expression were visible in the bridge site of E2-treated mice 10 days after injury (Figure 1(b)). At 12 days following injury, few MBP signals were observed in the control nerve bridge, whereas more MBP signals were observed in E2-treated mice (Figure 1(b)). The same result was obtained through western blot for MBP using nerve bridge tissue collected 12 days after injury (Figure 3(b)). At 15 days after injury, expression of MBP in the control group became stronger but was still less compared with that of the hormone-treated group (Figure 1(b)). At 30 days after injury, level of expression of MBP in the control nerve bridge was almost the same as that of the hormone-treated samples (Figure 1(b)). To determine whether E2 influenced axon growth from the proximal stump, MBP was costained with neurofilament using SMI-31R antibody. However, no significant difference between the control and hormone-treated nerve bridges was found (Figures 1(b) and 1(c)), indicating that estrogen does not influence extension of axon in the nerve bridge.

**Figure 2:**
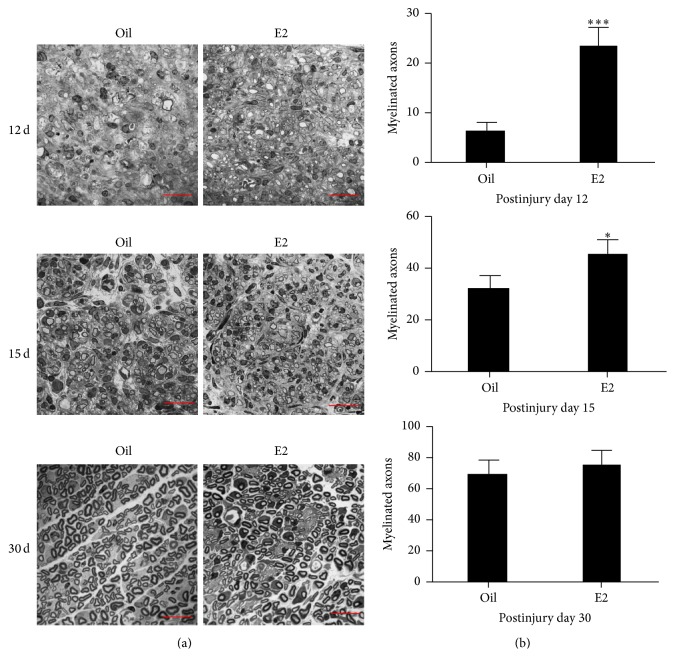
E2 treatment accelerated early myelin formation in the nerve bridge site upon sciatic nerve transection. (a) Toluidine blue staining of the transverse semithin sections of the nerve bridge from injured sciatic nerves of control and E2-treated mice to illustrate myelin sheaths. Scale bars represent 25 *μ*M (1000x). (b) Quantification of myelinated axons per 2500 *μ*M^2^, based on (a) (*n* = 5; ^*∗*^ *P* < 0.05, ^*∗∗∗*^ *P* < 0.001).

The toluidine blue-stained semithin transverse slice of the nerve bridge was observed to confirm whether E2 promoted early remyelination in the nerve bridge. More myelinated axons were found in hormone-treated mice compared with the control mice 12 and 15 days after injury, which is similar to the expression of MBP. However, the difference disappeared up to 30 days after injury (Figure 2).

### 3.2. E2 Upregulated AKT/mTOR Signaling in Schwann Cells

**Figure 3:**
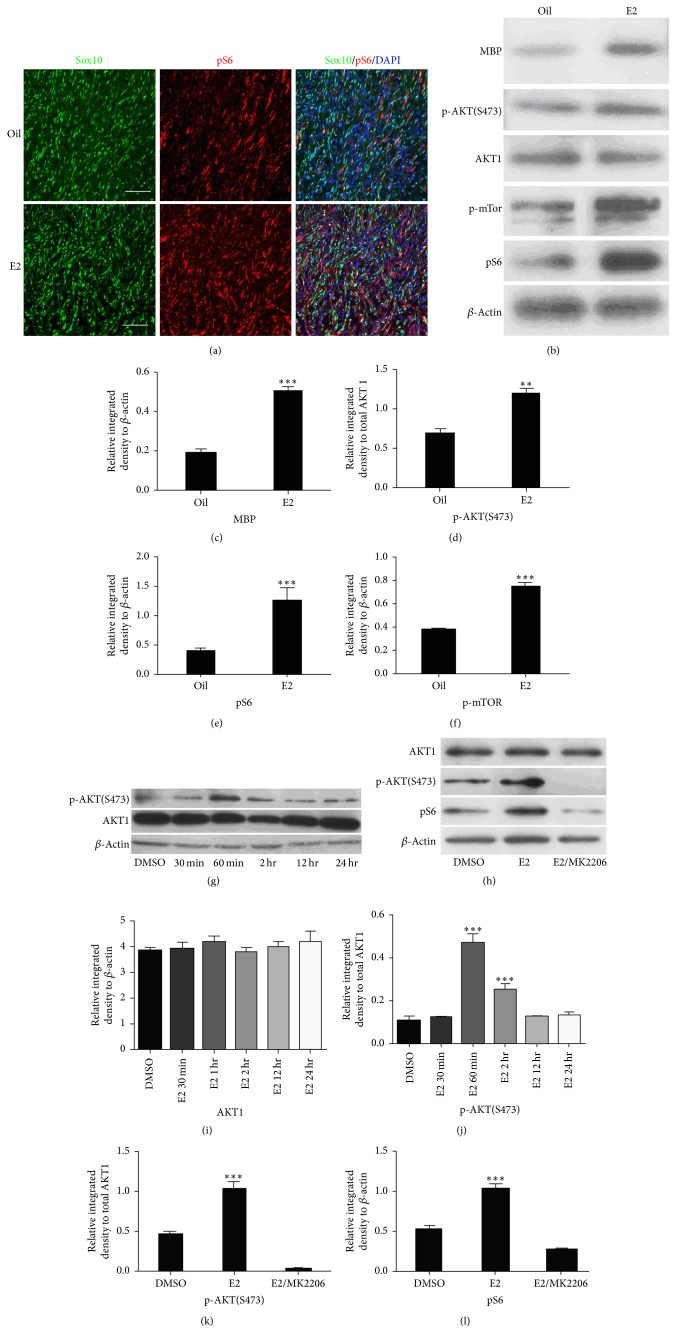
E2 treatment upregulated AKT/mTOR signaling in Schwann cells. (a) Immunostaining for the expression of pS6 (red signal) in the longitudinal section of the nerve bridge from the mouse sciatic nerve 12 days after injury. Sox10 (green signal) was costained to illustrate Schwann cell linage cells. More pS6-positive cells were detected in the E2-treated nerve bridge than in the control. Scale bars represent 60 *μ*M (400x). (b) Western blot analysis for MBP, total AKT, p-AKT (S473), pS6, and p-mTOR from tissue lysates to study AKT/mTOR signaling in nerve bridge site. The western blot analysis of the nerve bridge tissue also revealed higher phosphorylated AKT (p-AKT), pS6, and p-mTOR levels in the hormone-treated mice than in the control mice. (c–f) Quantification of the relative expression intensities of MBP, p-AKT (S473), pS6, and p-mTOR calculated from (b) (*n* = 3; ^*∗∗*^ *P* < 0.01, ^*∗∗∗*^ *P* < 0.001). (g) Western blot analysis for total AKT and p-AKT (S473) expressions in primary Schwann cells treated with E2 for 0 min, 30 min, 60 min, 2 h, 12 h, and 24 h. (h) Western blot analysis for total AKT, p-AKT (S473), and pS6 expression in primary Schwann cells treated with E2, E2+MK2206, or DMSO for only 60 min. (i, j) Quantification of relative AKT and p-AKT (S473) expression intensities calculated from (g) (*n* = 3; ^*∗∗∗*^ *P* < 0.001, E2 60 min versus DMSO; E2 2 h versus DMSO). (k, l) Quantification of relative p-AKT (S473) and pS6 expression intensities calculated from (h) (*n* = 3; ^*∗∗∗*^ *P* < 0.001). AKT, protein kinase B; DMSO, dimethyl sulfoxide; MBP, myelin basic protein; NF, neurofilament; p-mTOR, mammalian target of rapamycin.

The phosphorylated ribosomal protein S6 (pS6), a downstream effector of mTOR, was stained in the nerve bridge to determine whether E2 also activates the AKT/mTOR signaling in Schwann cells. More pS6-positive cells were detected in the E2-treated nerve bridge than in the control (Figure 3(a)). The western blot analysis of the nerve bridge tissue also revealed higher phosphorylated AKT (p-AKT), pS6, and p-mTOR levels in the hormone-treated mice than in the control mice (Figures 3(b)–3(f)). Schwann cells do not contribute to all the cells in the nerve bridge; thus the AKT/mTOR signaling was also detected in the primary Schwann cells, and E2 was found to significantly increase the p-AKT level of Schwann cells 1 h after administration (Figures 3(g) and 3(j)), without influencing the total AKT1 level (Figures 3(g) and 3(i)). The increasing p-AKT level was accompanied by an increase in pS6 level 1 h after E2 administration, and the effect of E2 on AKT and S6 phosphorylation is completely blocked by MK2206, a highly selective AKT1, AKT2, and AKT3 inhibitor (Figures 3(h), 3(k), and 3(l)).

### 3.3. E2 Promoted Schwann Cell Precursor Proliferation in an AKT-Dependent Manner

**Figure 4:**
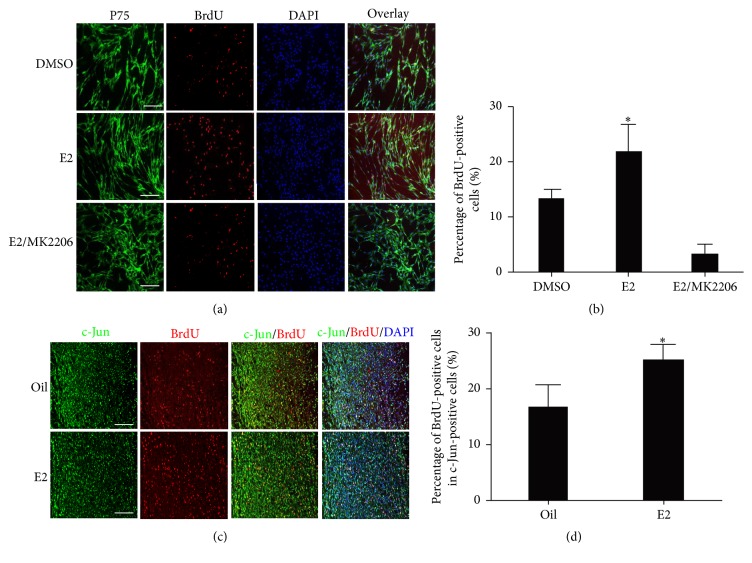
E2 treatment promoted Schwann cell proliferation. (a) BrdU staining (red signals) for primary Schwann cells treated with E2, E2+MK2206, or DMSO for only 24 h. P75 positive staining (green signals) was used to illustrate immature Schwann cells. Scale bars represent 50 *μ*M (400x). (b) BrdU-positive Schwann cell quantification from (a) (*n* = 5, ^*∗*^ *P* < 0.05, E2 versus DMSO). (c) BrdU staining (red signals) in the longitudinal section of the nerve bridge site in the injured sciatic nerve from E2-treated and control mice. The c-Jun was costained (green signals) to illustrate immature Schwann cells. Scale bars represent 100 *μ*M (200x). (d) BrdU-positive Schwann cell quantification from (c) (*n* = 5, ^*∗*^ *P* < 0.05). BrdU, 5-bromo-2′-deoxyuridine; DAPI, 4′,6-diamidino-2-phenylindole; DMSO, dimethyl sulfoxide.

Previous research showed that estrogen was a mitogen for primary Schwann cells when forskolin was in the medium of the cells [33]. However, its mechanism has not been discovered. The E2-promoted primary Schwann cell proliferation on adding forskolin was detected through the BrdU incorporation assay (Figures S2(A) and S2(B)). The mitogen potential of E2 on Schwann cell precursors (P75 positive) was found to have been completely blocked by the AKT inhibitor MK2206 (Figures 4(a) and 4(b)). The c-Jun and BrdU in the slice of nerve bridge site were costained to determine whether E2 also promotes Schwann cell proliferation in vivo because c-Jun is a transcription factor that is expressed in immature Schwann cells and contributes to nerve regeneration [36, 37]. The percentage of BrdU-positive cells in the c-Jun-positive cells was found to be greater in the E2-treated mice than in the control mice. The cell proliferation marker Ki67 was also costained with Schwann cell lineage marker Sox10 to confirm the above results, and the percentage of Ki67-positive cells in the Sox10-positive cells was also found to be greater in the E2-treated nerve bridge (Figures S2(C) and S2(D)).

### 3.4. E2 Promoted Differentiation and Myelination of Schwann Cell In Vitro in an AKT-Dependent Manner

**Figure 5:**
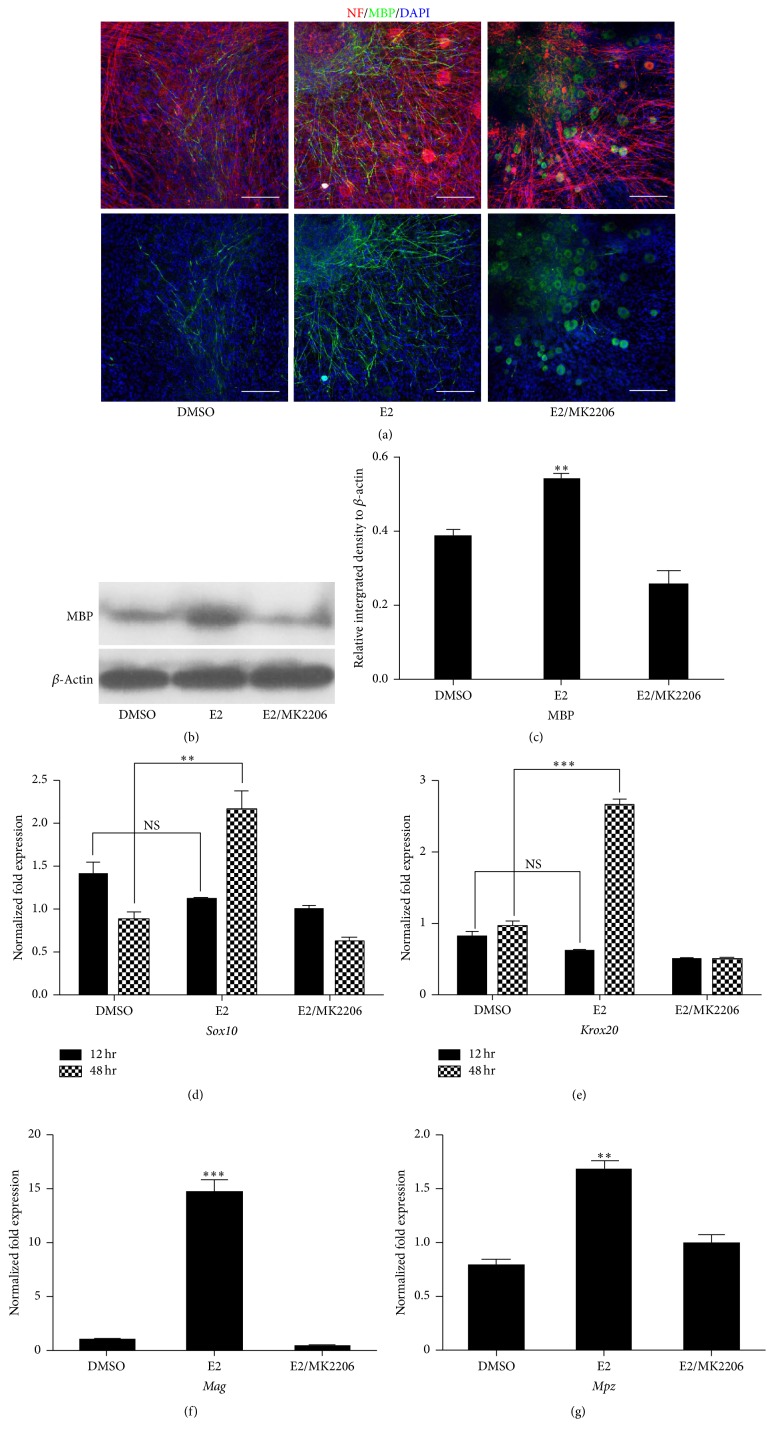
E2 promotes Schwann cell differentiation and myelination in vitro. (a) MBP staining (green signals) of myelinated DRG explant culture treated with E2, E2+MK2206, or DMSO for 1 week to detect myelin segments, and NF staining (red signals) was used to illustrate axons. Scale bars represent 100 *μ*M (200x). (b) Western blot analysis of MBP expression in the DRG explant culture. (c) Quantification of the relative MBP expression intensity based on the results of (b) (*n* = 3, ^*∗∗*^ *P* < 0.01, E2 versus DMSO). (d, e) Relative Sox10 (*n* = 4, *P* < 0.01, E2 48 h versus DMSO 48 h; NS, E2 12 h versus DMSO 12 h) (d) and Krox20 (*n* = 4, *P* < 0.001, E2 48 h versus DMSO 48 h; NS, E2 12 h versus DMSO 12 h) (e) mRNA expressions in primary SCs treated with E2, E2+MK2206, or DMSO for 24 h and 48 h, measured using qPCR. (f, g) Relative Mag (*n* = 4, *P* < 0.001, E2 versus DMSO) (f) and Mpz (*n* = 4, *P* < 0.01, E2 versus DMSO) (g) mRNA expression in primary SCs treated with E2, E2+MK2206, or DMSO for 48 h, measured using qPCR. DMSO, dimethyl sulfoxide; DRG, dorsal root ganglion; MBP, myelin basic protein; qPCR, quantitative polymerase chain reaction. ^*∗∗∗*^ *P* < 0.001.

A previous study showed that demyelination is induced by adding forskolin after myelin forms in the DRG-Schwann cell coculture system, remyelination begins when forskolin is eliminated, and E2 inhibits demyelination and promotes remyelination in this in vitro myelin injury model [32]. To investigate whether E2 promotes the initial myelination in vitro, the DRG explant culture was used in this study to illustrate myelination in vitro. When added simultaneously with ascorbic acid to the culture for a duration of one week to allow sufficient in vitro myelination, E2 was found to increase the expression of MBP in the culture via both immunofluorescence and western blot analysis assay (Figures 5(a)–5(c)). To investigate the mechanism of E2 in promoting Schwann cell myelination further, the mRNA level of* Sox10* and* Krox20*, key transcription factors for Schwann cell maturation and myelination, was measured via quantitative reverse transcription-polymerase chain reaction in E2-treated primary Schwann cells. The E2 treatment upregulated the expression of both transcription factors 48 h after administration and upregulated* Mag* and* Mpz* (two of the components of the myelin sheath) expressions. This indicated that E2 promoted Schwann cell maturation in vitro (Figures 5(d)–5(g)). The promoting effect of E2 on Schwann cell promyelination gene expression and myelination in vitro was completely blocked by MK2206 (Figures 5(a)–5(g)).

## 4. Discussion

Estrogen is a neuroprotective factor in several injuries and degeneration diseases in the CNS. Estrogen also regulates demyelination and remyelination in the CNS by acting on oligodendrocytes [26, 28, 30, 31] and may contribute to the better prognosis of women than men in MS [13, 14]. However, little is known about the effect of estrogen on Schwann cells and peripheral nerve regeneration. The present study found that E2 promotes early remyelination in the “nerve bridge” site after mouse sciatic nerve transection, possibly due to the positive effects of E2 on Schwann cell proliferation and differentiation. Similar to the results of the previous studies, estrogen promoted Schwann cell proliferation in vitro in a medium with forskolin [33].The in vitro E2 promotes Schwann cell proliferation in the presence of forskolin but promotes Schwann cell differentiation without forskolin. The treatment with E2 also promotes Schwann cell myelination in a coculture with DRG neurons. The effects of E2 on Schwann cell proliferation and differentiation depend on AKT activation.

Previous in vitro studies demonstrated that estrogen promotes Schwann cell proliferation [33] and protects it from hydrogen peroxide-induced cell death. Estrogen also inhibits demyelination and promotes remyelination in DRG-Schwann cell cocultures [32]. However, whether estrogen promotes peripheral nerve remyelination after injury is still unclear. A previous study [11] reported that a “nerve bridge” was formed within 5 days after sciatic nerve transection in rat. In this study, the sciatic nerve transection mouse model showed that E2 promotes Schwann cell proliferation and early remyelination in the nerve bridge site formed between the proximal and distal stumps, a position not subject to Wallerian degeneration, after nerve transection. Therefore, the effect of estrogen on peripheral nerve remyelination after mechanical injury was shown more accurately in this study. This study also demonstrates that administration of E2 also promotes Schwann cell proliferation and myelination in vitro, which might contribute to the effect of estrogen on the in vivo remyelination in mice.

Ras/ERK and PI3K/AKT pathways are activated in the Schwann cell. The effects of these pathways on peripheral nerve have been well established, such that the former inhibits Schwann cell myelination and promotes demyelination, whereas the latter promotes myelination and enhances remyelination. These are not always true because the ERK1/2 deletion in Schwann cell precursors disrupts the differentiation and marked hypomyelination of axons, whereas the cell-specific ablation of laminin gamma1 causes apoptosis, prevents Schwann cell proliferation, and hypomyelinates the peripheral nervous system (PNS) in an AKT-dependent manner [38]. ERK and AKT pathways are involved in forskolin-induced primary Schwann cell proliferation [39]. The AKT pathway is required for Schwann cell proliferation and the early stages of myelination in the coculture system [19]. The above studies suggest that the same signaling pathway plays different functions in regulating Schwann cell phenotypes, depending on certain cellular environments or developmental stages. Previous studies have shown that E2 rapidly activates AKT signaling in oligodendrocytes in vitro [26], and selective estrogen receptor *β* agonists activate PI3K/AKT/mTOR signaling in oligodendrocytes in vivo [28]. In this study, it was shown that estrogen activated the AKT/mTOR signaling in Schwann cells and promoted both Schwann cell proliferation and myelination in vitro in an AKT-dependent manner, which is consistent with the previously mentioned dual effects of AKT signaling on Schwann cells [26, 39]. Enough Schwann cells must be generated by proliferation for bridge formation, axon guidance, and sequential remyelination when in contact with newly generated axons. Thus, the estrogen-AKT pathway possibly affects the different stages of Schwann cells during nerve regeneration and induces both proliferation and differentiation on the Schwann cells in the nerve bridge. The effect of E2 on ERK phosphorylation in Schwann cells was determined in this study because ERK signaling is important for Schwann cell proliferation, myelination, and demyelination. Only a slight increase in p-ERK1 level was found in the nerve bridge tissue from E2-treated mice (Figures S3(A) and S3(B)) and in the primary Schwann cells (Figures S3(C) and S3(D)) via western blot analysis. This reaction interestingly seemed to be AKT dependent because AKT inhibitor MK2206 treatment inhibited ERK1 phosphorylation, suggesting that crosstalk between AKT and ERK pathways exists upon treatment with estrogen.

Several transcription factors for Schwann cell maturation and myelin component expression have been identified, among which Krox20 is specifically expressed in premyelinating Schwann cells and initiates myelin component transcription, whereas Sox10 is expressed in Schwann cells of different developmental stages and directly initiates myelin component transcription and indirectly promotes myelin gene transcription by promoting the expression [40]. Previous studies have shown that progesterone upregulates* Krox20* mRNA expression within 1 h of treatment in primary Schwann cells, whereas estrogen does not [17, 18]. The upregulation of* Krox20 *by progesterone is followed by upregulation of* Sox10* expression within 2 h [41], suggesting that progesterone directly induces the expression of* Krox20 *via the genomic effect of its receptor. Late upregulation of* Sox10* and* Krox20 *mRNA expression was demonstrated in this study 48 h after the administration of E2, accompanied by upregulation of* Mpz *and* Mag* expression, suggesting a slow nongenomic pathway for the estrogen receptor in regulating such gene expressions and Schwann cell maturation. Estrogen promoted Schwann cell myelin gene expressions in an AKT-dependent manner, as expected.

However, estrogen is not the only factor that activates AKT signaling in Schwann cells. Signaling from ECM such as laminin-integrin signaling also activates the AKT pathway in Schwann cells despite the axonal Nrg1 [21, 38]. Denervated Schwann cells were found to express Nrg1 transiently as an autocrine/paracrine signal that promotes their own differentiation and remyelination [42]. A previous study has shown that estrogen is a mitogen for primary Schwann cells when forskolin is the medium of the cells [33]. However, its mechanism has not been discovered. In this study, it was shown that estrogen enhanced the AKT pathway to serve as a proliferation signal when forskolin was added, which increases intercellular cAMP, but with axonal Nrg1, estrogen enhanced the AKT pathway to serve as a differentiation signal without forskolin.

Estrogen exerts its biological function binding to the estrogen receptor (ER). ER*α* and ER*β* are known as the classic ER and can mediate both genomic and nongenomic effects of estrogen. E2 also has nonclassic membrane associated receptors among which GPR30 is the one most studied [43]. In the CNS, activation of ER*β* may promote remyelination of oligodendrocytes in the mouse model of MS via upregulation of AKT/mTor signaling [28, 44]. In addition, activation of GPR30 was also found to improve CNS remyelination by oligodendrocytes [45]. Previous studies demonstrated that primary SCs expressed both ER*α* and *β* [46]. By immunostaining assay we found that primary SCs expressed GPR30 as well (data not shown). However, which type of ER contributes to the activation of AKT/mTor signaling and regulation of remyelination in Schwann cells by E2 has not been studied yet. In the future, we hope to figure out this issue through use of specific ER agonist and antagonist or other loss and gain of function experiments.

In conclusion, this study demonstrates the function of estrogen in protecting from peripheral nerve injury and partly reveals its mechanism. The E2 was found to promote early remyelination in the “nerve bridge” site after mouse sciatic nerve transection, possibly due to the positive effects of E2 on Schwann cell proliferation and differentiation. The in vitro E2 promoted Schwann cell proliferation in the presence of forskolin but promoted Schwann cell differentiation without forskolin. The E2 treatment also promoted Schwann cell myelination in a coculture with DRG neurons. The effects of E2 on Schwann cell proliferation and differentiation depend on AKT activation. These results indicate the potential therapeutic effect of estrogen on peripheral nerve injury.

## Supplementary Material

In the supplementary materials, we provided to readers some additional experimental data to increase the credibility of this research. In Figure S1, we showed the process of the “nerve bridge” formation, and the morphological difference between “nerve bridge” site and the distal stump site of the injured nerve. In Figure S2, we provided to the readers more proofs that E2 promotes Schwann cells proliferation in vitro and in vivo. Finally in Figure S3, we demonstrated the regulating role of E2 on the ERK signaling in Schwann cells.
