# Supplementary material for: 17β-Estradiol Promotes Schwann Cell Proliferation and Differentiation, Accelerating Early Remyelination in a Mouse Peripheral Nerve Injury Model
Source: Biomed Res Int. 2016 Oct 30;2016:7891202. doi: 10.1155/2016/7891202 (PMC5107215; doi:10.1155/2016/7891202)

Supplementary figures

Fig S1. Nerve bridge formed after sciatic nerve transection in mouse.

A. Observation of nerve bridge formation in longitudinal section of transectioned mouse SNs by an ordinary stereomicroscope without any staining (DS: distal stump; NB: nerve bridge; PS: proximal stump; SN, sciatic nerve).

B. A photo of SN at the 10th day after transection shows a myelin lacking nerve bridge site, which looked more transparent, and myelin-bearing distal stump (DS) and proximal stump (PS) on both sides of the nerve bridge (NB), which looked white and opaque.

C. Toluidine blue staining of transverse semi-thin sections of intact SN, nerve bridge at post day 10 and distal stump at post day 10 and 15. Scale bars represent 100 μM.

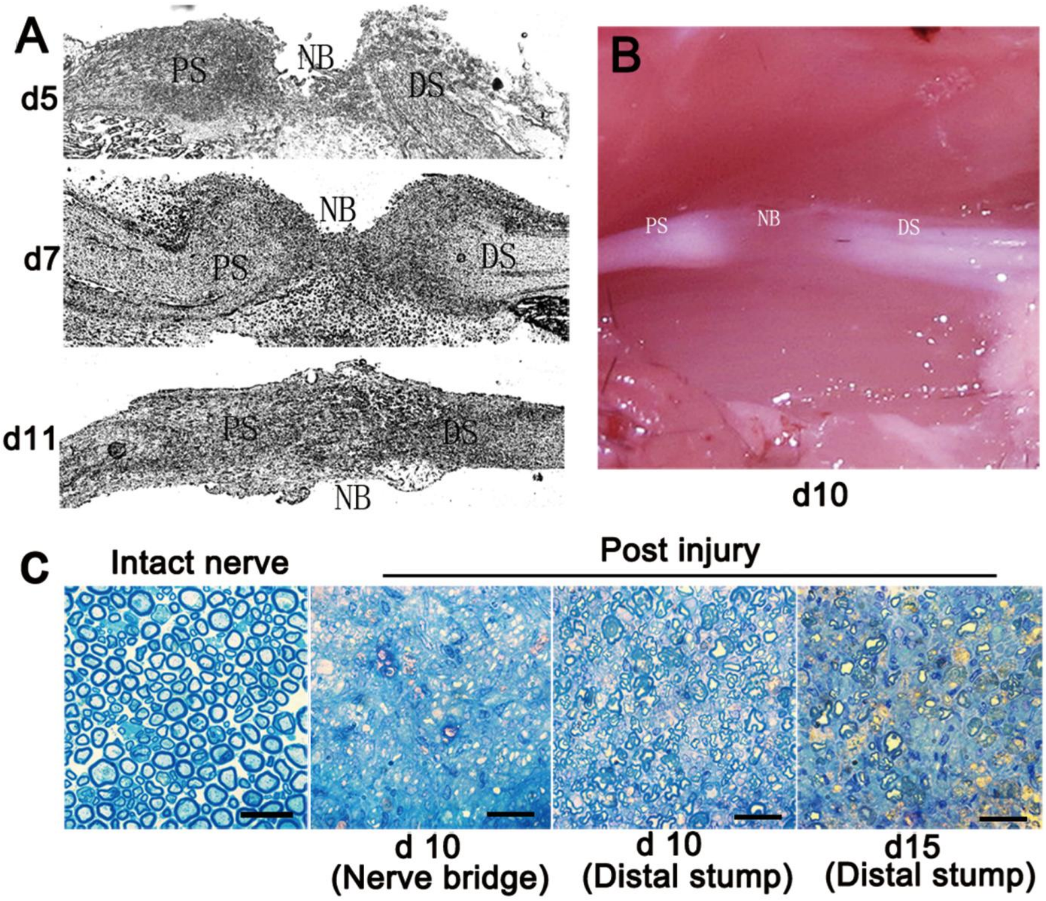

A
d5
PS
NB
DS
d7
NB
PS
DS
d11
PS
DS
NB
B
PS
NB
DS
d10
C
Intact nerve
Post injury
d 10
(Nerve bridge)
d 10
(Distal stump)
d15
(Distal stump)

Fig S2. 17β-estradiol promoted Schwann cell proliferation.

A. BrdU staining (red signals) for primary Schwann cells treated with E2or DMSO for 24 h in culture medium without β1-heregulin but with forskolin. S100β co-staining (green signals) was used to illustrate Schwann cells. Scale bars represent 100 μM (200×).

B. Quantification of BrdU-positive Schwann cells from C (n=5, ***P*<0.01).

C. Ki67 staining (red signals) in the longitudinal section of nerve bridge site in injured Sciatic nerve from E2-treated and control mice.Sox10 was co-stained (green signals) to illustrate Schwann cell lineage cells. Scale bars represent 100 μM (200×).

D. Quantification of BrdU-positive Schwann cells from E (n=5, **P*<0.05). BrdU, 5-bromo-2'-deoxyuridine; DMSO, dimethyl sulfoxide.

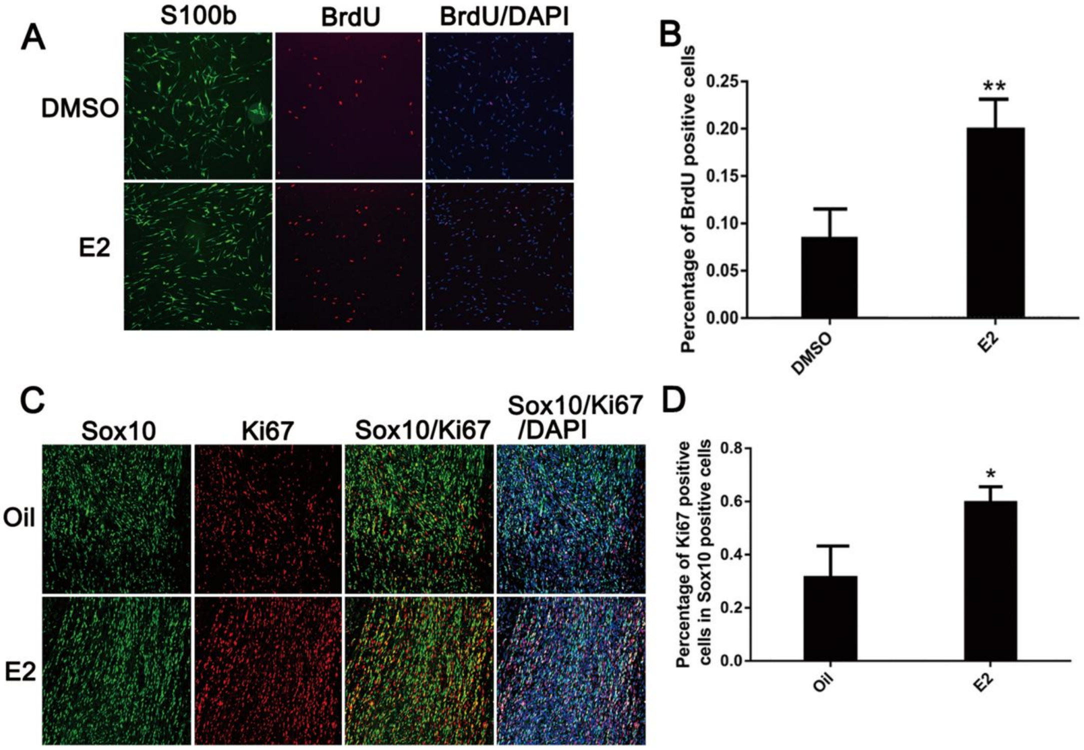

Fig S3. 17β-estradiol promoted Erk phosphorylation in dependence of AKT pathway.

A. Western blotting of Erk1 and phosphorylated Erk1 (p-Erk1) in nerve bridge tissue lysate.

B. Quantification of the relative intensity of p-Erk1 expression based on A (n=3).

C. Western blotting of Erk1 and p-Erk1 expression in primary SCs treated with E2, E2+MK2206 or DMSO for different duration.

D. Quantification of the relative intensity of p-Erk1 expression based on C (n=3, *P<0.05 E2 vs DMSO; **P<0.01 E2 vs Oil). DMSO, dimethyl sulfoxide; SC, sciatic nerve.

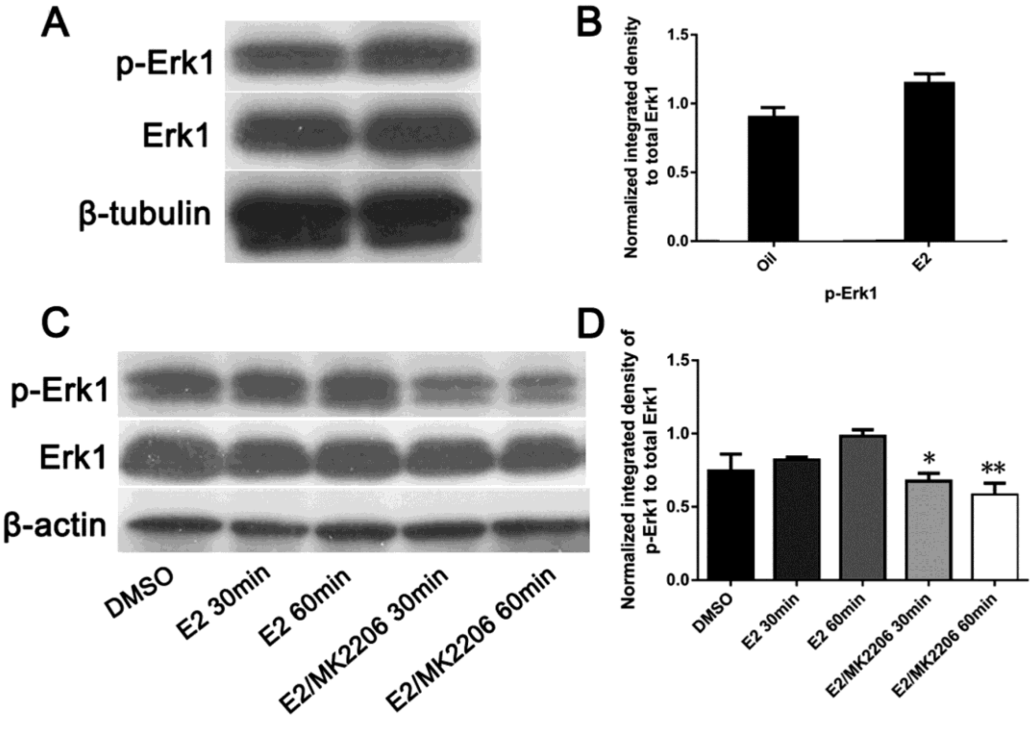

Supplement: Supplementary file 1 — In the supplementary materials, we provided to readers some additional experimental data to increase the credibility of this research. In Figure S1, we showed the process of the “nerve bridge” formation, and the morphological difference between “nerve bridge” site and the distal stump site of the injured nerve. In Figure S2, we provided to the readers more proofs that E2 promotes Schwann cells proliferation in vitro and in vivo. Finally in Figure S3, we demonstrated the regulating role of E2 on the ERK signaling in Schwann cells. [file 7891202.f1.pdf]
